# Nanomaterial-Based Carbon Paste Electrodes for Voltammetric Determination of Naproxen in Presence of Its Degradation Products

**DOI:** 10.1155/2019/5381031

**Published:** 2019-04-16

**Authors:** Hassan A. M. Hendawy, Waheed M. Salem, Mahmmoud S. Abd-Elmonem, Elmorsy Khaled

**Affiliations:** ^1^National Organization for Drug Control and Research (NODCAR), P.O. Box 29, Cairo, Egypt; ^2^Chemistry Department, Faculty of Science, Damanhour University, Damanhour, Egypt; ^3^Microanalysis Laboratory, Applied Organic Chemistry Department, National Research Centre, El Bohouth St., Dokki, 12622 Giza, Egypt

## Abstract

The present work describes a novel, simple, and fast electroanalytical methodology for naproxen (NAP) determination in pharmaceutical formulations and biological fluids in the presence of its degradation products. Carbon paste electrodes (CPEs) modified with different carbon nanomaterials, namely, glassy carbon powder (GCE), multiwall carbon nanotubes (MWCNTs), single-walled carbon nanotubes (SWCNTs), graphene nanosheets (Gr), and graphene oxides (GO) were tested. Comprehensive studies were performed on the electrode matrix composition including the nature of the pasting liquids, pH, carbon nanomaterials, and mode of electrode modification. Two anodic oxidation peaks were recorded at 0.890 and 1.18 V in 1 × 10^−1^ mol·L^−1^ phosphate buffer solution at pH 6. Oxidation of naproxen (NAP) is an irreversible diffusion-controlled process. Calibration plots were rectilinear in the concentration ranging from 0.067 to 1.0 *µ*g·mL^−1^ with correlation coefficient 0.9979. Photodegradation of NAP resulted in disappearance of the oxidation peak at 1.18 V, allowing simultaneous determination of NAP in presence of its degradation product. The achieved high sensitivity and selectivity suggest the application of the proposed protocol for naproxen determination in pharmaceutical preparations and human blood plasma.

## 1. Introduction

Naproxen, 2-(6-methoxynaphthalen-2-yl) propanoic acid, is a nonsteroidal anti-inflammatory drug (NSAID) commonly used for the treatment of moderate and severe pain, fever, inflammation, and stiffness [1, 2] through inhabitation of both COX-1 and COX-2 enzymes that cause inflammation and pain in the body [3]. Moreover, NAP is suggested in case of rheumatoid arthritis and other inflammatory rheumatic diseases [4, 5].

Determination of NAP in pharmaceutical formulations and biological fluids has been proposed by applying spectrophotometric [6, 7], spectrofluorimetric [8, 9], capillary isotachophoretic [10], and high-performance liquid-chromatographic methods coupled with spectrophotometric [11], amperometric [12], or mass spectrometric detectors [4].

The aforementioned techniques are demandingly laborious and time-consuming and require ancillary instrumentation. Samples prior to derivatization and the use of organic solvents are some of the inherent disadvantages. Electrochemical methods are effective tools for the determination of pharmaceutical compounds as they are faster, cheaper, easier, and more sensitive than spectrometric and HPLC methods. Several reports and comprehensive reviews about voltammetric methods for quantification of drugs have been found in the literature [13–17].

Naproxen has been determined voltammetrically by applying mercury electrodes for the cathodic reduction of NAP [18]; however, mercury electrodes have some limitations as they are toxic and there is rapid deterioration of the electrode response. Alternatively, Pt electrodes were suggested for anodic oxidation of naproxen [19]; the high background current with limited sensitivity and reproducibility due to contamination by the fouling products and impurities was reported [20]. Recently, other electrode materials were applied including boron-doped diamond [21, 22], gold electrode [23], glassy carbon [24], graphite electrode [25, 26], multiwall carbon nanotube-modified glassy carbon electrode (MWCNTs/GCE) [27, 28], and carbon paste electrodes modified with different nanomaterials [29–31] were reported.

Herein, the differential pulse voltammetric protocol for determination of naproxen in pharmaceutical formulations and biological fluids in presence of its degradation product using carbon paste electrodes modified with different nanomaterials has been suggested.

## 2. Experimental

### 2.1. Reagents and Chemicals

Ultrapure water with electric resistivity ∼18.3 MΩ·cm (Milli-Q system, Millipore) was used for preparing supporting electrolytes and stock solutions. Britton–Robinson buffer was prepared, and the desired pH value was adjusted with the appropriate amount of 2 × 10^−1^ mol·L^−1^ NaOH solution.

Graphite powder (synthetic 1-2 mm, Aldrich) or glassy carbon powder (GC, “Sigradur-G” type, HTW Meitingen, Germany) was used for electrode fabrication. Different carbon nanaomaterials including multiwall carbon nanotubes (MWCNTs, Aldrich), single-walled carbon nanotubes (SWCNTs, Aldrich), graphene nanosheets (Gr, Sigma), and graphene oxides (GO, Sigma) were tested. Paraffin oil (PO; Merk, Germany), silicone oil (SO; Sigma Aldrich), or tricresyl phosphate (TCP, Fluka) were applied as pasting liquids.

### 2.2. Authentic Samples

An authentic sample of naproxen (C_14_H_14_O_3_, 230.259 g·mol^−1^) was obtained from the National Organization for Drug Control and Research, Giza, Egypt. The stock drug solution (1 × 10^−4^ mol·L^−1^) was freshly prepared by dissolving the appropriate amounts of NAP in 10^−2^ mol·L^−1^ NaOH solution.

### 2.3. Pharmaceutical Preparation

Naprosyn tablets (250 mg NAP/tablet; Egyptian Group for Pharmaceutical Industries, Cairo, Egypt) were purchased from local drug stores. One tablet was grinded and dissolved in 50 mL NaOH solution. Naproxen content was assayed according to the proposed and HPLC methods [32].

### 2.4. Biological Samples

Aliquots of the biological fluid (plasma, obtained from a healthy male) were spiked with different NAP concentrations, treated with 0.1 mL of 70% perchloric acid diluted to 10 mL, vortexed for 1.0 min, and centrifuged for 10 min at 13000 rpm. The supernatants were neutralized with NaOH to the appropriate pH value, and the volume was completed to 25 mL with water.

### 2.5. Apparatus

All voltammetric experiments were carried out using a Metrohm computrace voltammetric analyzer model 797 VA with software version 1.0 (Metrohm, Switzerland) equipped with Ag/AgCl (3 mol·L^−1^ KCl) and platinum electrodes as reference and auxiliary electrodes, respectively. The pH measurements were carried out using a 692 pH meter (Metrohm, Herisau, Switzerland) with a combined pH glass electrode (6.0202.100).

The working carbon paste electrodes were prepared by mixing 0.5 g of carbon materials (either graphite powder or GC) with 0.2 g of paraffin oil in a ceramic mortar for 15 min. Alternatively, 10% of carbon powder was replaced with different nanomaterial, and the paste was prepared by the same manner. Homogenous carbon pastes were pushed into the individual Teflon piston holders with conductive electric wires for electric contact with a potentiostat [33]. Electrode surface regeneration was performed via polishing with a wet filter paper.

For surface modification, 20 *µ*L of the nanomaterial suspension (2 mg·mL^−1^ in DMF) was drop-casted on inverted CPEs (graphite/PO matrix) and left to dry for 24 h at 25°C. The modified CPEs were rinsed with deionized water and used directly in the electrochemical cell.

### 2.6. Procedures

An appropriate volume of the NAP stock solution was added to 15 mL of Britton–Robinson buffer at the desired pH value. The voltammograms were recorded using differential pulse voltammetry (DPV), with the following parameters: pulse height, +50 mV; pause before scan, 2 s; pulse width, 100 ms; pulse time, 40 ms; and scan rate, 40 mV·s^−1^.

## 3. Results and Discussion

### 3.1. Electrochemical Oxidation of Naproxen

**Figure 1:**
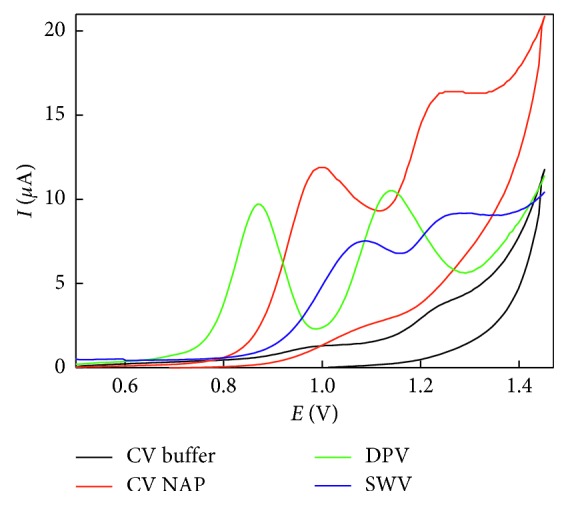
Voltammetric behavior of naproxen on the carbon paste electrode surface in 2 × 10^−1^ mol·L^−1^ phosphate buffer solution at pH 6. The scan rate employed was 50 mV·s^−1^ and NAP concentration 3.0 *µ*g.

The electrochemical behavior of NAP on blank carbon paste electrodes was evaluated by applying cyclic voltammetry and differential pulse voltammetry at pH 6 (Figure 1). Under CV conditions, the anodic peak at 0.999 V corresponds to naproxen oxidation with the formation of an intermediate carboxylic radical, followed by decarboxylation. The second anodic peak appeared at 1.249 V corresponding to another current maximum that possibly belongs to ketone (2-acetyl-6-methoxynaphthalene, AMN), which in agreement with more recent voltammetric studies discussed two one-electron transfers in oxidation of NAP in aqueous media [24, 26, 34, 35]. No cathodic peaks were recorded indicating the irreversibility of naproxen oxidation over the carbon paste electrode, the working electrode.

Differential pulse voltammetry showed sharp and well-defined peaks at 0.976 and 1.221 V with an improved peak height compared with CV; therefore, further studies related to the quantitative determination of naproxen were carried out using the DPV technique. It is noteworthy to mention that the square wave voltammetric technique showed two peaks at 1.06 and 1.25 V but with lower peak height compared with DPV.

### 3.2. Optimization of Electrode Matrix Compositions

The experimental conditions were optimized for the NAP electrochemical response in order to achieve the highest analytical performance. Thus, the effect of electrode matrix compositions, pH of the supporting electrolyte, and electrochemical parameters were studied and optimized.

#### 3.2.1. Effect of Pasting Liquid and Working pH Range

NAP is considered as a NSAID of the propionic acid group with a pKa value of 4.15 [36]. At higher pH values, naproxen is completely deprotonated and thus an uncharged radical would be formed during the first electrochemical oxidation process, followed by decarboxylation [27]. The second one-electron transfer may involve the formation of a cation that is stabilized by the methoxynaphthyl ring through resonance structures.

**Figure 2:**
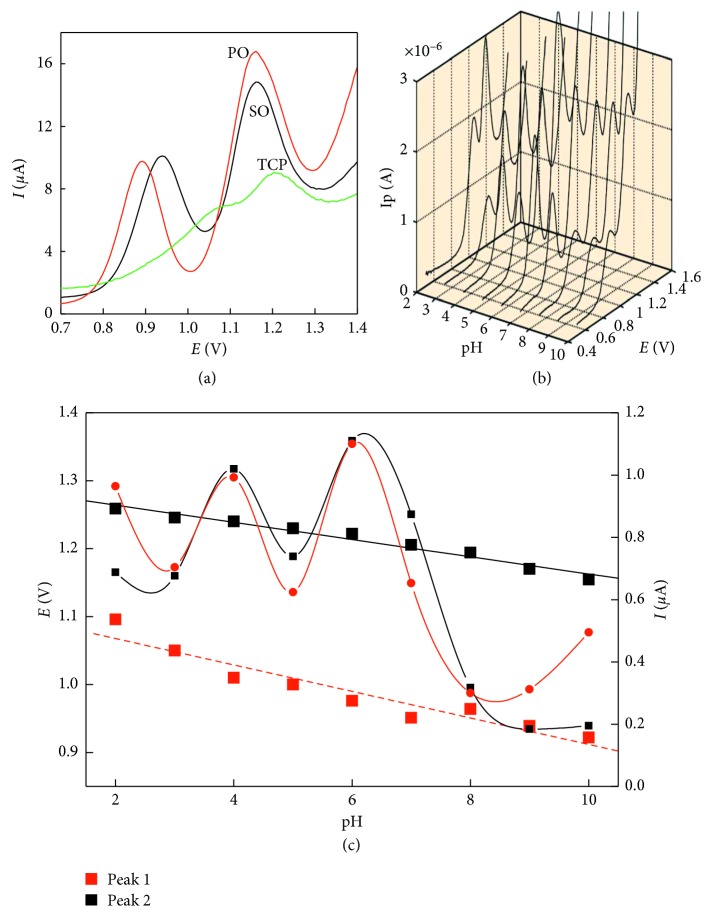
Differential pulse voltammograms for 4.5 *µ*g NAP (a) using different pasting liquids, (b) at different pH values, and (c) peak potential and peak currents at different pH values. The scan rate employed was 50 mV·s^−1^.

Silicon oil showed a wide working pH range from 3 to 8 with the optimum at pH 4 (S1). On the contrary, the peaks obtained using TCP as a pasting liquid were very poor and will be ignored in the following experiments (Figure 2(a)).

Application of the paraffin oil as pasting liquids showed improved peak heights with the shift of the second oxidation peak by about 50 mV compared with silicon oil-based electrodes. Moreover, the electrochemical oxidation of NAP at CPE/PO electrodes was investigated at different pH values ranging between 2.0 and 10.0 (Figure 2(b)). The peak potentials (*E*_p_) of both NAP oxidation peaks were shifted toward less positive oxidation potentials via increasing pH value, indicating the electrode reaction is governed by a proton transfer process. The relationship between the peak potential and the pH value of the solution was evaluated as *E*_p1_ (V) = 1.2895–0.0127 pH, *r* ^2^ = 0.9694 and *E*_p2_ (V) = 1.1068–0.0195 pH, *r* ^2^ = 0.9143 (Figure 2(c)). The maximum peak height of both oxidation peaks was recorded at pH 6, which will be selected for the following studies.

#### 3.2.2. Nanomaterial Impact on Sensor Performance

*(1) Bulk-Modified Electrodes*. Nanostructure-modified electrodes have been adopted as a promising approach to facilitate the direct electron transfer of biomolecules and improving the electrode performance. Herein, the traditional graphite powder usually applied for fabrication of carbon paste electrodes was completely replaced by glassy carbon powder (GC) and MWCNTs or graphene. Alternatively, 10% incorporation of nanomaterials with the carbon paste matrix was also tested.

**Figure 3:**
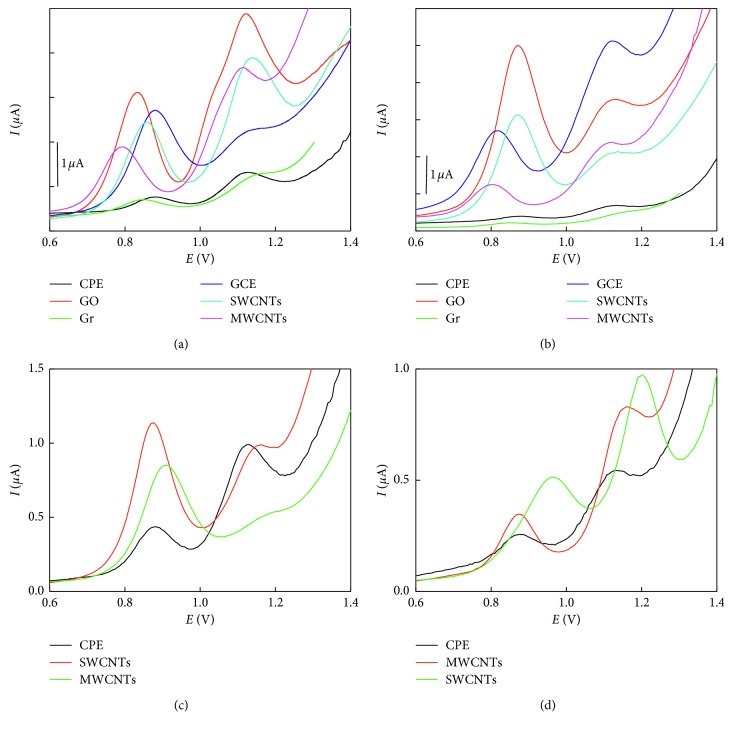
Differential pulse voltammograms of NAP using carbon paste electrodes modified in bulk and surface with different nanomaterials (a, c) dissolved in NaOH and (b, d) dissolved in methanol. The scan rate employed was 50 mV·s^−1^.

Using NaOH as a solvent for NAP, carbon paste electrodes showed two irreversible oxidation peaks for NAP at 0.880 and 1.14 V, respectively (Figure 3(a)). Glassy carbon powder-based electrodes showed better performance compared with graphite/CPE. However, complete replacement with MWCNTs or graphene showed complete disappearance of the oxidation peaks which may be attributed to the high hydrophobicity of the carbon nanotubes.

Different performances were achieved by replacing 10% of the graphite powder with different nanomaterials (Figure 3(a)). SWCNT-incorporated electrodes showed the best performance with two well-defined peaks at 0.856 and 1.14 V, respectively. Even graphene oxide (GO) provides improved first oxidation peak at 0.833 V; the second one at 1.2 V was broader than that of SWCNTs. Similar to graphene oxide, MWCNTs showed a well-defined peak at lower oxidation potential (0.790 V) with an improved peak height compared with CPE and a broad peak at 1.11 V.

On application of methanol as solvent for NAP (Figure 3(b)), the tested materials showed different behavior where graphene oxide was the best followed by SWCNTs and glassy carbon powder. The first oxidation peak was recorded at 0.869, 0.875, 0.821, and 0.803 for GO, SWCNTS, GC, and MWCNTs, respectively.

The effect of the nanomaterial within the electrode matrix was investigated by varying the SWCNT content from 2.5 to 20%, and 10% was the most promising.

*(2) Surface-Modified Electrodes*. Based on the previous notification of the effect of carbon nanomaterials, an alternative approach for improving the electrode performance can be attributed via modification of the working electrode surface by drop-casting of nanomaterial solution in DMF (2 mg·mL^−1^) on the electrode surface. After drying and evaporation of the organic solvent, thin layers of the carbon nanomaterials were deposited on the electrode surface on which the oxidation of the target analyte takes place.

According to the voltammetric peaks represented in Figure 3(c), SWCNTs showed the best performance compared with other nanomaterials tested. In NaOH medium, no noticeable shifting in the peak potential was observed, while in methanol, SWCNTs showed higher peak height with shifting toward more positive potential.

The remarkable enhancement in current response and shifting of the peak potential provide clear evidence of the catalytic effect of the nanomaterial-modified carbon paste electrode which acts as a promoter to enhance the electrochemical reaction, considerably accelerating the rate of electron transfer. Indicative of a mass transport regime that includes a thin-layer diffusional process (entrapment of naproxen species within the carbon nanotube film) is presented as a possible explanation for the lowered oxidation potential and substantial current increase. Moreover, the methoxynaphthyl ring in naproxen may interact strongly with the carbon nanotube structure through *π*–*π* bonds [37, 38]. Therefore, instead of only considering the change of mass transfer regime of NAP species, this strong interaction may contribute for the voltammetry profile and higher current increase for NAP presented.

#### 3.2.3. Electrode Surface Poisoning

**Figure 4:**
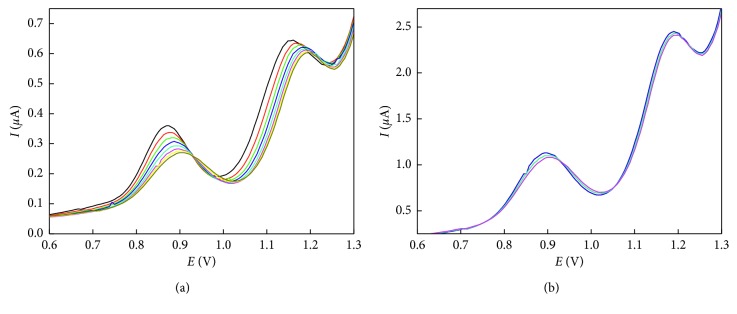
Differential pulse voltammograms for 8 consecutive measurements of 3 *µ*g NAP in phosphate buffer at pH 6 using (a) carbon paste electrodes and (b) SWCNTs/CPE. The scan rate employed was 50 mV·s^−1^.

The voltammetric recording for consecutive measurements of NAP on the native carbon paste electrode resulted in a constant decrease in current and shifting of both oxidation peaks toward more positive potential, which may be attributed to the adsorptive properties of NAP or its oxidation products on the electrode surface (Figure 4(a)). Modification with nanomaterials enhances the electrode performance and diminishes the electrode poisoning, which indicates that carbon nanotubes provided antifouling properties probably due to faster electron kinetics (Figure 4(b)).

In agreement with these results, it is established the hypothesis that some of the naproxen oxidation products become adsorbed at the electrode surface [19, 21, 24] and that through controlled oxidation, it is possible to “saturate” the surface of the electrode, thus obtaining constant signals that improve the repeatability of analyses.

### 3.3. Effect of Scan Rate

**Figure 5:**
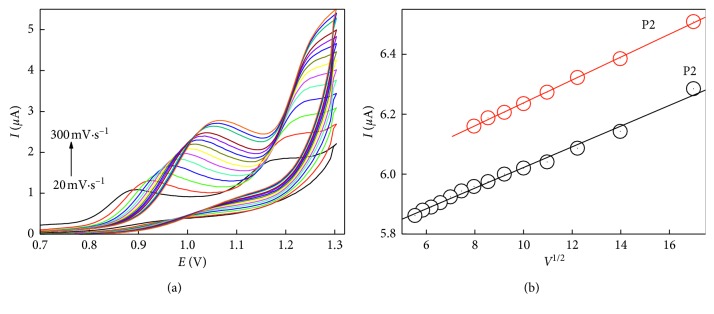
Effect of scan rate on the voltammetric behavior of 3.0 *µ*g NAP using SWCNTs/CPE in 2 × 10^−1^ mol·L^−1^ phosphate buffer pH.

Oxidation of naproxen was carried out at different scan rates ranging between 20 and 300 mV·s^−1^ (Figure 5). The peak potential and the variation of the anodic current maxima with the square root of the scan rate were plotted. Linear trend of both oxidation peak currents of NAP vs the root of scan rate (*r* ^2^ was 0.999) on the working electrode surface was obtained, concluding that the naproxen oxidation process is controlled by diffusion of the electroactive species toward the electrode surface [39], which was reported in a previous report using the MWCNT-modified GCE for NAP detection [27].

### 3.4. Determination of Naproxen in Presence of Its Degradation Product

Marotta et al. [40] reported the photodegradation of naproxen 1-(6-methoxy-2-naphthyl) ethanol and 2-acetyl-6-methoxynaphthalene and conversion of the carboxylic group to ethanol and finally ketone, which results in absence of the second oxidation peak. The obtained results were elucidated by performing the IR spectra of NAP and its degradation product (S2).

**Figure 6:**
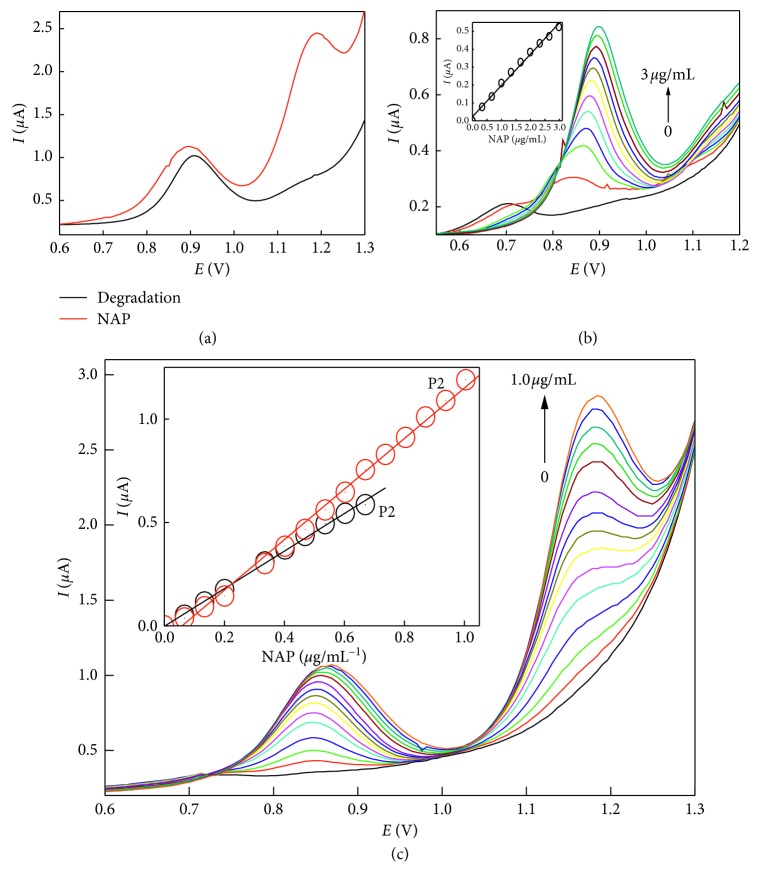
Differential pulse voltammograms of naproxen and its degradation product on SWCNTs/CPE.

Naproxen showed two oxidation peaks at 0.85 V that correspond to naproxen oxidation with the formation of an intermediate carboxylic radical, followed by decarboxylation at 1.18 V due the formation of ketone (2-acetyl-6-methoxynaphthalene, AMN). Upon photodegradation and formation of AMN, only the first oxidation peak at 0.890 V with the disappearance of the second peak was achieved, allowing the simultaneous voltammetric determination of naproxen in presence of its degradation product (Figure 6(a)). The calibration graph for the degradation product was linear in the concentration range 0.067 to 0.73 *µ*g·mL^−1^ with similar slope values to the NAP (Figure 6(b) and Table 1).

At the optimum measuring conditions applying carbon paste electrodes incorporated with 10% SWCNTs, the standard calibration curves using SWCNT-modified carbon paste electrodes were performed. The peak current at 0.85 V increased linearly with increasing the NAP in the concentration range 0.067 to 0.67 *µ*g·mL^−1^, while performing calibration at 1.18 V, the calibration graph was linear up to 1.0 *µ*g·mL^−1^ with more reproducibility and sensitivity.

The linearity with regression parameters was calculated according to ICH guidelines (Table 1). The high values of correlation coefficient (*r*) and low values of standard deviation (SD), standard error (SE), showed the assemblage of the points around the calibration graph and proved the linearity of the method over the specified concentration range.

### 3.5. Analytical Applications

According to the obtained results, it was possible to apply this technique to the quantitative analysis of NAP in pure form, dosage form, and plasma. The proposed method was successfully applied for the determination of NAP in its pharmaceutical dosage form (Naprosyn tablets; 250 mg NAP/tablet) using SWCNTs/CPEs.

The results obtained by the proposed method were compared with those obtained from the reported method [32] according to *t*-test and *F*-test (Table 2).

To validate the suggested procedure, the linearity, range, limit of detection, limit of quantification, accuracy, and robustness were measured according to the ICH guidelines.

## 4. Conclusion

In this study, it was shown that carbon paste electrodes modified with carbon nanomaterials can be considered as a sensitive working electrode for simultaneous voltammetric determination of naproxen in presence of its degradation product. From the different method for electrode fabrication, bulk modification with SWCNTs in the PO/CPE showed an effective electrocatalytic activity toward the anodic oxidation of naproxen, which leads to a great increase in the peak current (more than 8-fold). The present study showed comparable sensitivity with previously published NAP sensors (Table 3) with the advantage of simultaneous determination of NAP in presence of its degradation product. The LODs were achieved by the CNT-modified electrode for NAP allowing application of such method for determination of NAP in biological samples without further pretreatment.

## Figures and Tables

**Table 1:** Regression and statistical parameters obtained from differential pulse voltammetry calibration curves of naproxen and its degradation product using SWCNTs/CPE.

Parameters	NAP		Degradation product
	At 0.85 V	At 1.18 V	
Concentration range (*µ*g·mL^−1^)	0.067–0.67	0.067–1.00	0.067–0.73
Slope of regression line (*μ*A)	0.9083	1.2264	0.7696
*s*_a_ (*μ*A)	0.0154	0.0321	0.0096
Intercept of regression line (b) (*μ*A·mL·*μ*g^−1^)	0.0021	−0.0779	0.0141
*s*_b_ (*μ*A·mL·*μ*g^−1^)	0.00626	0.0194	0.0043
Correlation coefficient (*r*)	0.9988	0.9956	0.9993
LOD (*µ*g·mL^−1^)	0.1042	0.0812	0.045
LOQ (*µ*g·mL^−1^)	0.31572	0.2461	0.135
SD	0.023	0.025	0.015
RSD (%)	3.04	2.78	3.46

**Table 2:** Application of the proposed and reference method for the determination of NAP in pure form, dosage form, and plasma.

Parameters	Pure form		Dosage form		Plasma	
Method	Proposed method^*∗*^	Reference method [32]^*∗*^	Proposed method^*∗*^	Reported method [32]^*∗*^	Proposed method^*∗*^	Reported method [32]^*∗*^
% found	102.14	101.2	100.17	100.8	96.54	97.9
	100.45	100.65	100.22	99.7	97.32	99.0
	99.9	99.8	99.6	98.8	98.0	99.2
Mean ± S.D.	100.83 ± 1.67	100.55 ± 0.71	99.99 ± 0.34	99.77 ± 1.0	97.28 ± 0.73	98.7 ± 0.7
*t*-test	0.922 (2.776)		0.876 (2.776)		0.842 (2.776)	
*F*-test	2.345 (19)		1.512 (19)		1.344 (19)	

^*∗*^Each result is the average of three different separate determinations. Figures in parentheses are the tabulated *t* and *F* values, respectively, at *P*=0.05.

**Table 3:** Comparison of analytical parameters of different naproxen electrodes.

Working electrode	Electrochemical technique	Linear range (*µ*M)	LOD (*µ*M)	Sample	Degradation product	Reference
Glassy carbon electrode	DPV	10–125	0.3	Tablets	No	[24]
Boron-doped diamond	DPV	0.5–50	0.03	Tablets	Yes	[21]
Platinum electrode	DPV	4.03–108	1.04	Tablets	No	[19]
ZnO/MWCNTs/CPE	SWV	1.0–200	0.23	Tablets	No	
MWCNTs-Gr-Il/GCE	DPV	1–100	0.125	Blood plasma	No	[31]
MWCNTs/GCE	Amperometry	10–100	0.6	Tablets	No	[27]
Graphite electrode	DPV	4.9–123	4.45	Tablets	No	[25]
SWCNTs/CPE	DPV	4.35–65.5	6.255	Tablets and blood plasma	Yes	Present work

## Data Availability

The data used to support the findings of this study are available from the corresponding author upon request.

## Supplementary Materials

Supplementary Materials S1: differential pulse voltammograms for 10.0 × 10^–6^ mol·L^−1^ NAP using silicon oil carbon paste electrodes at different pH values. S2: FT-IR spectra of naproxen and its degradation product.
