# Supplementary material for: Nanomaterial-Based Carbon Paste Electrodes for Voltammetric Determination of Naproxen in Presence of Its Degradation Products
Source: J Anal Methods Chem. 2019 Apr 16;2019:5381031. doi: 10.1155/2019/5381031 (PMC6501153; doi:10.1155/2019/5381031)
Supplement: Supplementary Materials — S1: differential pulse voltammograms for 10.0 × 10–6 mol·L−1 NAP using silicon oil carbon paste electrodes at different pH values. S2: FT-IR spectra of naproxen and its degradation product. [file 5381031.f1.docx]

|  |
| --- |
| **S1: Differential pulse voltammograms for 10.0 ×10^-6^ mol L^-1^ NAP using silicon oil carbon paste electrode at different pH values.** |

|  |
| --- |
| **S2: FT-IR spectra of naproxen and its degradation product** |
